# Editorial: COVID-19: psychopathology of a pandemic

**DOI:** 10.3389/fpsyt.2024.1448701

**Published:** 2024-07-19

**Authors:** Iván Echeverria, Marc Peraire, Marcelo O’Higgins, Ana Benito, Gonzalo Haro

**Affiliations:** ^1^ TXP Research Group, Universidad Cardenal Herrera-CEU, CEU Universities, Castellón de la Plana, Spain; ^2^ Department of Mental Health, Consorcio Hospitalario Provincial de Castellón, Castellón de la Plana, Spain; ^3^ Department of Psychiatry, School of Medical Sciences, National University of Asunción, Asunción, Paraguay; ^4^ Torrente Mental Health Unit, Hospital General Universitario de Valencia, Torrente, Spain

**Keywords:** biopsychosocial model, COVID-19, diathesis stress model, epidemiological psychiatry, mental health, pandemic, psychopathology, psychiatric epidemiology

*I am myself and my circumstance, and if I do not save it, I do not save myself.*–José Ortega y Gasset, Spanish philosopher and essayist.

Human history is inextricably linked to the various plagues, outbreaks, epidemics, and pandemics that have been responsible for major social, demographic, cultural, economic, and political changes (1). However, epidemiology has not only written our past, but it continues to shape the present through contemporary infectious epidemiological events (IEE). Traditionally, the focus in an IEE has been on the pathogen, its spread and the threat it poses to physical health. However, after the SARS (2002) and MERS (2012) epidemics, mental health began to be taken into account, and with the arrival of COVID-19 (2019) it was noted that alongside the epidemiological pandemic there could be a pandemic of psychopathology (2).

These events would lead to a new concept and field of psychiatry: Epidemiological Psychiatry (EP). EP studies the impact, distribution and variety of mechanisms by which an IEE can produce psychopathology in the population (3). It considers that the development of psychopathology during an IEE parallels the epidemiological process of outbreaks, epidemics and pandemics: just as these have their origin in a pathogen that is transmitted and disseminated through vectors and ends up causing the disease, psychopathology has its origin in fear, uncertainty and misinformation; it is transmitted by social isolation, the media and economic instability; and finally, due to an overload of resilience and adaptation mechanisms, results in entities such as anxiety, depression, acute stress disorder, post-traumatic stress disorder (PTSD), substance use disorders or burnout in predisposed individuals (1).

Based on Engel’s Biopsychosocial Model (BPS) (4), EP considers that the different elements related to an IEE can be identified as social, biological and psychological factors specific to each individual, and that the interaction between these factors is responsible for health and disease processes. Thus, the imbalance between the nosological and salutogenic factors of the BPS, either by excess or defect, would configure a latent diathesis that could be activated by a stressor (such as a pandemic) and ultimately lead to psychopathology (5, 6).

In line with the ideas of Paterson et al., where cross-outbreak approach is proposed to assess stigma, EP aims to provide a holistic, cross-IEE framework to address the multitude of factors and processes involved in the development of psychopathology, focusing on individual conditions to understand the heterogeneity of mental health outcomes at the group level (7). At the same time, it is worth distinguishing EP from Psychiatric Epidemiology, which studies the aetiology and prevalence of mental disorders in society.

Thus, using the EP framework as a beacon, this Research Topic aims to shed light on the various biological, psychological, and social factors that may have influenced the development of psychopathology during the COVID-19 pandemic.

Among the biological factors, some studies included in this Research Topic have shown the effect of age and sex, with being female and young being a risk factor for developing psychopathology during the pandemic (Fountoulakis et al.). In turn, being male and young implied a greater risk of presenting craving for substances of abuse and therefore a shorter duration of abstinence (Daigre et al.). In this sense, Sorokin et al. observed that in patients with COVID-19 serum/plasma inflammatory markers are associated with the manifestation of psychopathological symptoms. Regarding genetics, another study Zihao et al. used genome-wide significant SNPs in depression, anxiety, and different COVID-19 statuses and analyzed them through Mendelian randomization to evaluate the relationship between them. On the other hand, brain imaging has also been used to analyze the changes that have occurred at the brain level during COVID-19, observing in Popovich et al. a reduction over time of brain activations related to fear-associated learning tasks during the pandemic period, and their associations with post-traumatic stress symptoms.

Regarding psychological factors, history of mental disorders or the presence of comorbid personality traits was associated with an increased risk of psychopathology and substance use during the pandemic (Regzedmaa et al.; Daigre et al.). On the other hand, the influence of stress on mental health was a hot Research Topic during COVID-19, as shown by Lu et al. Specifically, its effects on anxiety, depression, or resilience were the most popular areas of concern. Precisely, several studies conducted during the pandemic addressed resilience-related dimensions, such as purpose in life, moral courage, and character strengths, which modify the occurrence of psychopathology (8, 9). Moreover, these dimensions also impacted on burnout development that, in turn, was another psychological factor involved in the development of psychopathology, especially on healthcare workers (10, 11). Indeed, the relationship between burnout and psychopathology was reciprocal and direct, with more burnout having more psychopathology and vice versa (10).

Concerning social factors, the idiosyncrasy of each country may have influenced the prevalence of psychopathology during the pandemic. In this sense, an interesting study by Fountoulakis et al. analyzed the mental health of students in 11 countries, and Gichangi et al. did so in a country as little studied as Kenya. Finally, Villalobos and Hernandez-Rodriguez focused on the particular situation of Latinx adults living on the US-Mexico border, thus deepening the impact of sociocultural differences on mental health in the COVID-19 context. In addition, gender roles have also influenced in the mental health during the pandemic, with women having more depressive symptomatology, as showed in Navarro et al. At the same time, occupation has been another relevant point in the development of mental disorders, as seen in healthcare workers from Austria (Santillan-Ramos et al.) or Indonesia (Maruf et al.). Moreover, the evolution of the pandemic and related elements such as national lockdowns, peaks in infection rates or greater financial worries influenced the temporal dynamics of mental health, as shown in a five-wave longitudinal study by Weber et al. Also in a longitudinal way, Martinez-Torteya et al. deepened in the socioeconomic conditions and psychological status evolution of university students in Mexico. However, the persistence of psychopathology due to the COVID-19 pandemic remains to be seen.

These findings reinforce the relevance and vision of EP, and demonstrate the public health issue that mental health represents during an IEE. At the same time, this concept supports that in future risk scenarios, health policies should be promoted to address psychopathology preventively rather than palliatively. Ultimately, it would accelerate and optimize risk assessment, diagnosis and treatment of mental disorders, which would mean applying the concept of precision and personalized psychiatry to IEE.

## Author contributions

IE: Writing – original draft, Writing – review & editing. MP: Writing – review & editing. MO: Writing – review & editing. AB: Writing – review & editing. GH: Writing – review & editing.
